# Calcium-Sensing Receptor as a Novel Target for the Treatment of Idiopathic Pulmonary Fibrosis

**DOI:** 10.3390/biom15040509

**Published:** 2025-04-01

**Authors:** Kasope Wolffs, Renjiao Li, Bethan Mansfield, Daniel A. Pass, Richard T. Bruce, Ping Huang, Rachel Paes de Araújo, Bahareh Sadat Haddadi, Luis A. J. Mur, Jordanna Dally, Ryan Moseley, Rupert Ecker, Harry Karmouty-Quintana, Keir E. Lewis, A. John Simpson, Jeremy P. T. Ward, Christopher J. Corrigan, Renata Z. Jurkowska, Benjamin D. Hope-Gill, Daniela Riccardi, Polina L. Yarova

**Affiliations:** 1 School of Biosciences, Cardiff University, Cardiff CF10 3AX, UK; lir54@cardiff.ac.uk (R.L.); mansfieldb@cardiff.ac.uk (B.M.); daniel@compassbioinformatics.co.uk (D.A.P.); brucert@cardiff.ac.uk (R.T.B.); huangp8@cardiff.ac.uk (P.H.); jurkowskar@cardiff.ac.uk (R.Z.J.); riccardi@cardiff.ac.uk (D.R.); 2 Department of Life Sciences, Aberystwyth University, Aberystwyth SY23 3DA, UK; rachel.paes@unesp.br (R.P.d.A.); bah13@aber.ac.uk (B.S.H.); lum@aber.ac.uk (L.A.J.M.); 3 Molecular Oncology Laboratory, Experimental Research Unit, Faculty of Medicine, São Paulo State University (UNESP), Botucatu 18618-687, SP, Brazil; 4 School of Dentistry, Cardiff University, Cardiff CF14 4XY, UK; dallyj2@cardiff.ac.uk (J.D.); moseleyr@cardiff.ac.uk (R.M.); 5 TissueGnostics, 1020 Vienna, Austria; rupert.ecker@tissuegnostics.com; 6 School of Biomedical Sciences, Faculty of Health, Queensland University of Technology, Brisbane 4059, Australia; 7 The University of Texas Health Science Center at Houston, McGovern Medical School, Houston, TX 77030, USA; harry.karmouty@uth.tmc.edu; 8 Institute of Life Sciences, School of Medicine, Swansea University, Swansea SA2 8QA, UK; k.e.lewis@swansea.ac.uk; 9 Translational and Clinical Research Institute, Faculty of Medical Science, Newcastle University, Newcastle upon Tyne NE2 4HH, UK; j.simpson@newcastle.ac.uk; 10 King’s Centre for Lung Health, School of Immunology and Microbial Sciences, King’s College London, London SE1 9RT, UK; jeremyptward@gmail.com (J.P.T.W.); chris.corrigan@kcl.ac.uk (C.J.C.); 11 Department of Respiratory Medicine, Cardiff and Vale University Health Board, Cardiff CF14 4XW, UK; hope-gillb2@cardiff.ac.uk

**Keywords:** idiopathic pulmonary fibrosis, calcium/cation-sensing receptor, TGFβ1, arginine–polyamine pathway, negative allosteric modulator

## Abstract

Idiopathic pulmonary fibrosis (IPF) is a disease with a poor prognosis and no curative therapies. Fibroblast activation by transforming growth factor β1 (TGFβ1) and disrupted metabolic pathways, including the arginine–polyamine pathway, play crucial roles in IPF development. Polyamines are agonists of the calcium/cation-sensing receptor (CaSR), activation of which is detrimental for asthma and pulmonary hypertension, but its role in IPF is unknown. To address this question, we evaluated polyamine abundance using metabolomic analysis of IPF patient saliva. Furthermore, we examined CaSR functional expression in human lung fibroblasts (HLFs), assessed the anti-fibrotic effects of a CaSR antagonist, NPS2143, in TGFβ1-activated normal and IPF HLFs by RNA sequencing and immunofluorescence imaging, respectively; and NPS2143 effects on polyamine synthesis in HLFs by immunoassays. Our results demonstrate that polyamine metabolites are increased in IPF patient saliva. Polyamines activate fibroblast CaSR in vitro, elevating intracellular calcium concentration. CaSR inhibition reduced TGFβ1-induced polyamine and pro-fibrotic factor expression in normal and IPF HLFs. TGFβ1 directly stimulated polyamine release by HLFs, an effect that was blocked by NPS2143. This suggests that TGFβ1 promotes CaSR activation through increased polyamine expression, driving a pro-fibrotic response. By halting some polyamine-induced pro-fibrotic changes, CaSR antagonists exhibit disease-modifying potential in IPF onset and development.

## 1. Introduction

Progressive pulmonary fibrosis occurs in the setting of various interstitial lung diseases and is associated with increased mortality rates [1]. Idiopathic pulmonary fibrosis (IPF) has a worse 5-year survival rate (25%) than many cancers [2]. Treatments such as oxygen therapy and anxiolytics/opiates have been used to reduce breathlessness and cough [3], but currently, the only disease-modifying therapeutic options are the anti-fibrotic agents, pirfenidone and nintedanib [4]. These agents can slow declining lung function in some patients [5], but do not directly target IPF comorbidities [5,6] or significantly improve key patient-reported outcomes, such as quality of life [7], likely due to the common side effects [8,9]. There is currently no cure for IPF, and lung transplantation still represents the best therapeutic option, which restores lung function and improves survival [5,6]. However, most patients are precluded from transplantation due to their advanced age/frailty and the presence of comorbidities, such as pulmonary hypertension (PH) and lung cancer [5,6,10]. Thus, there is an urgent need to develop new disease-modifying therapeutics with fewer side effects.

The development of lung fibrosis involves the activation of multiple cell types and a complex network of signaling cascades, many of which are associated with G protein-coupled receptors (GPCR) [11]. Although the initiating events leading to IPF are poorly understood, the consensus is that the disease occurs in genetically susceptible individuals subjected to certain environmental stressors [12]. The lungs are exposed to the external environment, containing multiple harmful agents including, but not limited to, pathogenic microorganisms, toxic fumes, and pollutants. This interaction is thought to establish a continuous cycle of unresolved epithelial injury, stimulating a pro-fibrotic milieu, at the center of which is the cytokine, transforming growth factor beta (TGFβ), which is essential to the repair process, attendant fibroblast activation and excessive extracellular matrix (ECM) deposition [13,14].

In IPF, activated fibroblasts require an upregulation of key metabolic pathways to meet the demands of increased pro-fibrotic activity [15]. Specifically, the glutamine and arginine metabolism pathways are responsible for the increased expression of non-essential amino acids, glycine and proline, required for collagen synthesis, and polyamines such as putrescine and spermidine, which promote cell survival and growth [16,17,18]. These changes in metabolism are likely due to increased arginase expression or activity, which have been previously reported in human IPF lung tissue and the bleomycin model of mouse lung fibrosis [19,20].

The extracellular calcium/cation-sensing receptor (CaSR) is a member of the group C GPCRs. Named after its main physiological ligand, extracellular Ca^2+^ (Ca^2+^_o_), the CaSR is highly expressed in calcitropic organs (such as the parathyroid glands, kidneys, and bone), where it detects changes in the extracellular free ionized extracellular Ca^2+^ concentration ([Ca^2+^]_o_) [19,20]. CaSR is also expressed by non-calcitropic organs, including, but not limited to, the skin [21], vasculature [22], heart [23], and lungs [24], where it regulates differentiation, proliferation, inflammation, secretion, and contractility.

During fetal development, the CaSR promotes lung expansion by regulating fluid secretion and branching morphogenesis [25,26,27]. In the adult lung, the CaSR acts as a multi-modal chemosensor for components of environmental pollutants (e.g., Ni^2+^ and Cd^2+^), in addition to bacterial and viral infections (e.g., polyamines) [24,28,29]. CaSR is also activated by various endogenous polycations (e.g., eosinophil cationic protein, major basic protein), polyamines (e.g., putrescine, spermine, and spermidine), glutathione, and basic amino acids (e.g., L-arginine and L-ornithine), which are particularly abundant in the lung [28,30] and whose expression is increased in IPF [18].

CaSR activation leads to the induction of various signaling pathways associated with IPF pathogenesis, including increases in intracellular Ca^2+^ concentration ([Ca^2+^]_i_) [28] and Rho kinase-mediated actin stress fiber assembly [31], activation of ERK1/2 and PI3K/Akt [32,33], and a reduction in intracellular cyclic adenosine monophosphate (cAMP) concentrations [24]. Previously, CaSR signaling has been implicated in the proliferative and remodeling processes of diseases, such as asthma [24], cardiac fibrosis [22], pulmonary arterial hypertension (PAH) [34], and chronic obstructive pulmonary disease (COPD) [35], some of which are common IPF comorbidities [2,36,37].

This study investigates the involvement of polyamines in IPF pathogenesis and the ability of NPS2143, a CaSR-negative allosteric modulator (NAM), to suppress TGFβ1-induced pro-fibrotic effects in normal and IPF human lung fibroblasts (HLFs).

## 2. Methods

### 2.1. Human Studies

#### Flow Infusion Electrospray High-Resolution Mass Spectrometry (FIE-HRMS)

The study “Novel Technologies for Diagnosing and Monitoring Respiratory Diseases” obtained regional ethical approval from Hywel Dda University Health Board. This study was conducted in accordance with the Helsinki principles. Written informed consent was obtained from all participants. Eligibility included a diagnosis of IPF/UIP at the time of sample collection, which was determined at regional respiratory multidisciplinary team meetings. Control saliva samples were collected from spouses accompanying patients attending clinics with no IPF/UIP diagnosis.

FIE-HRMS was used to evaluate the potential of saliva as a source of non-invasive polyamine biomarkers for IPF. Spontaneous saliva was collected and processed from 6 patients with IPF and 6 control participants. The clinical characteristics of sample donors are summarized in Table S1.

Samples were processed using methanol and chloroform (4:1) and analyzed by FIE-HRMS, as described previously [38]. FIE-HRMS was performed using the Q Executive Plus Mass Analyzer Instrument with the UHPLC System (ThermoFisher Scientific, Bremen, Germany), where mass/charge (*m*/*z*) were generated in positive and negative ionization mode in a single run. All samples were run in duplicate with a blank of the reagents; no significant differences in the results were obtained. Metabolomic data were log-transformed with MetaboAnalyst (v4.0), using R (v3.5.1) and Bioconductor packages (v3.7). Statistical significance was assessed, and box plots were generated using GraphPad Prism 10.0.

### 2.2. In Vitro Studies

#### 2.2.1. Cell Culture

Primary human lung fibroblasts (HLFs) from healthy donors (CC-2612) were purchased from Lonza (Slough, UK).

Lung tissue samples from IPF patients undergoing lung transplantation were obtained from the International Institute for the Advancement of Medicine through collaborations with the UT Health Houston Pulmonary Center of Excellence biorepository led by Dr. Karmouty-Quintana (Houston, TX, USA). IPF primary human lung fibroblasts were isolated by explant outgrowth from cryopreserved distal airway-free lung tissue, i.e., parenchymal fibroblasts, according to published protocols [39,40].

HLFs were cultured in Lonza custom Fibroblast Growth Medium (FGM-2), containing basal medium (FBM) (CC-3131) and FGM SingleQuots supplements (CC-4126): 0.1% insulin, 0.1% human fibroblast growth factor-B (hFGF-B), 2% fetal bovine serum (FBS), and 0.1% gentamicin and amphotericin-B solution, according to the protocol provided by Lonza. Cells were maintained at 37 °C under humidified conditions in a 5% CO_2_ incubator, with media changes every 48–72 h.

#### 2.2.2. HLF Stimulation and Treatment

Once HLFs reached ~60% confluence, the growth medium was replaced with an experimental medium (FBM, 0.1% insulin, 0.1% hFGF-B, and 0.1% FBS) for 24 h. Next, the medium was changed to an experimental medium supplemented with one of the following treatments: (1) vehicle control: 0.01% dimethyl sulfoxide (DMSO, #D2650; Sigma-Aldrich, Merck, Dorset, UK); (2) CaSR negative allosteric modulator (NAM): treated with 1 µM NPS2143 (#ab145050; Abcam, Cambridge, UK); (3) TGFβ1: treated with 5 ng/mL TGFβ1 (#T7039; Sigma-Aldrich); (4) TGFβ1 + NAM: pre-treated with 1 µM NPS2143 for 4 h, followed by 5 ng/mL TGFβ1. Cells were maintained at 37 °C under humidified conditions in a 5% CO_2_ incubator for 72 h. Cells in the vehicle control group were used for the measurements of [Ca^2+^]_i_.

#### 2.2.3. Immunofluorescence Staining

HLFs were cultured using the protocol described above. Following treatment, cells were washed with PBS and fixed with 4% paraformaldehyde for 10 min at room temperature. The cells were permeabilized with 0.3% Triton X-100 for 10 min at room temperature. Non-specific antibody binding was prevented with blocking buffer (2% donkey serum and 1% bovine serum albumin (BSA) in phosphate-buffered saline (PBS) for 30 min. The cells were incubated with the relevant primary antibodies diluted in a blocking buffer at 4 °C overnight. Primary antibodies included mouse anti-human CaSR (5C10-ADD) monoclonal antibody, 1:500 (#ab19347; Abcam, Cambridge, UK); mouse anti-human αSMA monoclonal antibody, 1:500 (#ab7817; Abcam); or rabbit anti-human collagen I, 1:500 (#ab34710; Abcam). After washing with PBS, secondary antibodies raised in donkey (anti-mouse IgG AlexaFluor-488, 1:1000, #A21202 or anti-rabbit AlexaFluor-488, 1:1000, #A21206, ThermoFisher Scientific, Loughborough, UK) were applied for 1 h and incubated in the dark. After washing, cell nuclei were counterstained with DAPI (#62248; ThermoFisher Scientific) for 5 min and washed with PBS before mounting. Negative control staining was performed by omitting primary antibodies or replacing the CaSR antibody with an IgG2a isotype control (mouse, 1:100, #02-6200; ThermoFisher Scientific) (Figure S1). Images were captured using a Zeiss LSM 710 Confocal Fluorescence Microscope (Carl Zeiss Microscopy Ltd., Cambourne, UK).

#### 2.2.4. Quantitative Immunofluorescence Microscopy

Fluorescence intensities were quantified using StrataQuest (v7.0)^®^ (TissueGnostics GmbH, Vienna, Austria). Images were pre-processed to extract inputs using Gaussian smoothing, object boundary erosion, and background subtraction. To ensure only positively stained areas containing a nucleus were used for building measurement masks, nuclear object detection was performed first. This involved setting a mask to identify nuclei of approximately 42 µm^2^ from the DAPI channel of each image using the nuclear segmentation function within StrataQuest (v7.0). Measurement masks were then generated by overlaying the nuclei mask on the 488 nm channel of each image to permit the mask to “grow” in all directions, until encountering either a break in marker immunoreactivity or interaction with the measurement mask of an adjacent cell (maximum growing steps: 33 µm; skip steps: 0 µm), as illustrated in Figure S2. All objects and measurement masks were detected using thresholds, based on the greyscale values corresponding to the lowest and highest marker intensities confidently recognized as positive staining, with the observer blinded to the experimental conditions. Mean intensity values were calculated from greyscale fluorescence intensity values within the measurement masks for each image. All measurements were then normalized to the number of cells in that region of interest.

#### 2.2.5. Measurements of Intracellular Free Ionized Calcium Concentration ([Ca^2+^]_i_)

Single-cell fluorescence Ca^2+^ imaging experiments were performed using a monochromator-based fluorimeter system (OptoFluor; Cairn Research, Faversham, UK) and the ratiometric dye, Fura-2 Acetoxymethyl (Fura-2 AM) ester. All intensities were background-subtracted before the 340:380 nm emission ratio was used to calculate maximal fold-change from baseline. CaSR-induced changes in [Ca^2+^]_i_ were measured in Fura-2 loaded NHLFs, as described previously [24].

#### 2.2.6. RNA Sequencing

Total RNA was extracted and purified using the RNeasy Mini Kit, including the optional on-column DNase digestion (Qiagen, Manchester, UK), according to the manufacturer’s protocol. RNA was quantitated using a Qubit RNA BR Assay Kit and Qubit^®^ fluorometer (Fisher Fluorometer, ThermoFisher Scientific), and RNA integrity number (RIN) for each sample was measured using an Agilent 2200 TapeStation (Agilent Technologies, Stockport, UK), according to the manufacturer’s instructions. Only samples with RIN > 9.5 were used for library preparation.

Sequencing libraries were prepared using Illumina TruSeq Stranded Total RNA Kit (Illumina, Cambridge, UK). Dual Indexes were introduced using the Illumina Truseq RNA UD indexing plate to ensure accurate library sequencing. The resulting libraries were sequenced on the Illumina NextSeq 500 (Illumina) to generate 75 base-pair single-ended reads. A total of ~500 million reads were obtained across 12 samples. Each condition was sequenced in triplicate (different donors per replicate).

Reads were quality-checked with FastQC [41], trimmed with Trimmomatic [42], and aligned to the human reference genome version GRCh38.101 reference genome [43] using Spliced Transcripts Alignment to a Reference (STAR) package [44] version 2.7.3a. Duplicates were marked/removed using Picard (v2.12), and the raw reads were counted by FeatureCounts (v1.6.3) [45]. An additional quality control step was carried out using SortMeRNA (v4.0) [46], and differential gene expression was calculated using the SARTools (v1.7.3) pipeline [47] in DESeq2 (v1.24.0) [48]. ClusterProfiler (4.14.4) package in R (v3.5.1) was used to conduct the Kyoto Encyclopedia of Genes and Genomes (KEGG) enrichment analysis of differentially expressed genes (DEGs) [49,50,51,52]. The ReactomePA (1.50.0) package was performed to identify comprehensive pathway information [53]. *p* adjusted values (*p_adj_*) < 0.05 was used as a cut-off. Heatmaps were generated using the pheatmap (1.0.12) package in R (v3.5.1) [54].

The changes in gene expression were calculated for pairwise group comparisons, and the *p_adj_* values were used to account for the false discovery rate using the Benjamini–Hochberg correction. Quantification, statistical analysis, and figure generation were performed using R (v3.5.1) [55] and Prism 10 (GraphPad Inc., La Jolla, CA, USA).

#### 2.2.7. Polyamine Secretion

##### Ornithine ELISA

Ornithine was measured in supernatants of HLFs grown in culture using the competitive ELISA method, according to the manufacturer’s instructions (ImmuSmol, Bordeaux, France). Absorbance values were measured spectrophotometrically at a wavelength of 450 nm using a FLUOstar Omega Spectrophotometer Microplate Reader (BMG Labtech, Aylesbury, UK).

##### Spermine ELISA

Spermine was measured by using the competitive ELISA method in supernatants and cell lysates of HLFs, according to the manufacturer’s instructions (FineTest, Wuhan, China). Absorbance values were measured spectrophotometrically at a wavelength of 450 nm, as described above.

### 2.3. Statistics

Data were analyzed using the Prism 10 software package (GraphPad Inc.) and are expressed as the mean ± SD. Statistical comparisons were performed using the 2-tailed Student’s *t*-test or ANOVA (with Sidak’s post hoc analysis). *p* values of <0.05 were considered significant. *N* denotes the number of donors (biological replicates), while *n* represents the number of independent experiments (technical replicates). RNA sequencing data were analyzed using the R package, DESeq2 (v1.24.0). Adjusted *p* values (*p_adj_*) accounted for the false discovery rate using the Benjamini–Hochberg correction; the significance cut-off used for *p_adj_* values was 0.05. Quantification and statistical analysis were performed using R (v3.5.1) and Prism 10.

## 3. Results

### 3.1. Naturally Occurring Polyamines Are Enriched in the Salivary Metabolome of IPF Patients

**Figure 1:**
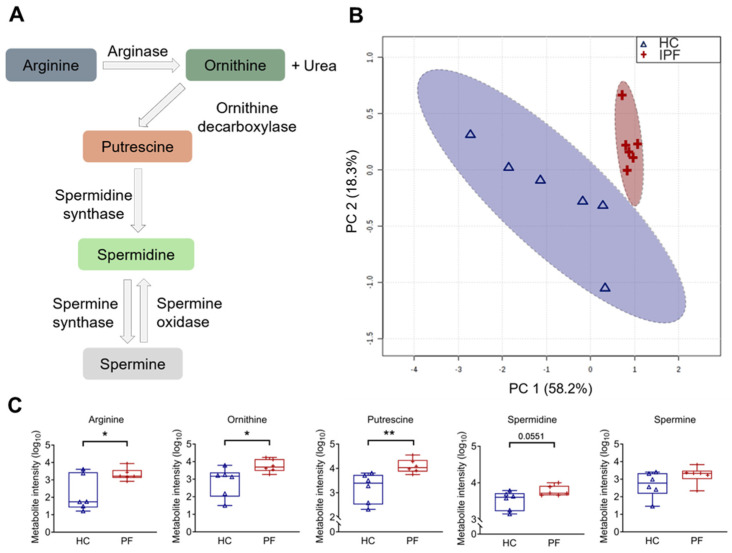
Arginine pathway metabolites are increased in idiopathic pulmonary fibrosis (IPF) patient saliva samples. (**A**) Diagram representing the arginine–polyamine metabolic pathway. (**B**) Principal component analysis (PCA) score plot between the main principal components (PC1 and PC2) shows a tight cluster of the IPF samples based on their metabolic profile compared to controls, with a 95% confidence ellipse drawn for each group (shown by the dotted lines). Assessment of PC1 and PC2, which account for 76.4% of the total variation between the control (HC) and IPF clusters, highlights the prominence of polyamines. (**C**) Associated box and whisker plots of metabolites in the arginine–polyamine pathway highlight the differences between healthy and IPF profiles. Y-axis: Log_10_ normalization of metabolite intensity. Statistical analysis was performed with unpaired two-tailed Student *t*-test; * *p* < 0.05, ** *p* < 0.01. *N* = 6 controls; 6 IPF patients.

Assessment of the variation between clusters indicated the prominence of the arginine–polyamine pathway in IPF (Figure 1A). Principal component analysis (PCA) on saliva samples demonstrated clear clustering of the IPF group, distinct from a more diffuse control group (Figure 1B). This result suggests a difference in the metabolome of IPF and controls. Plotting the loading vectors showed the importance of arginine and ornithine in driving the separation between the experimental groups; other polyamines were also relevant but showed smaller contributions (Figure S3). Box and whisker plotting of individual metabolites showed that IPF saliva samples contained elevated levels of arginine (*p =* 0.03), ornithine (*p =* 0.04), putrescine (*p =* 0.01), and spermidine (*p* = 0.06), compared with controls (Figure 1C). However, spermine enrichment levels were not significantly altered (*p* = 0.40).

### 3.2. CaSR Is Functionally Expressed in Normal Human Lung Fibroblasts (NHLFs)

**Figure 2:**
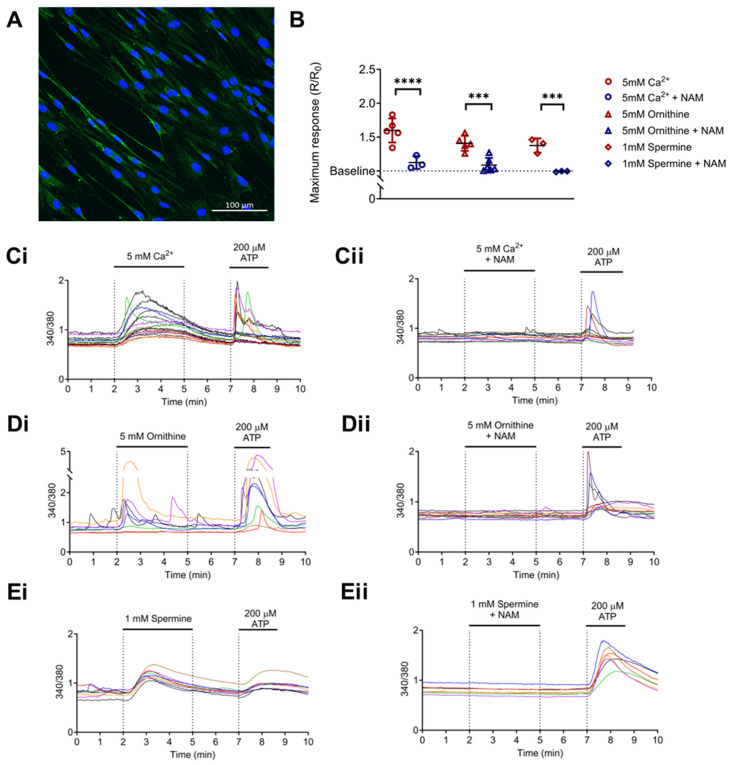
Calcium-sensing receptor (CaSR) is functionally expressed in normal human lung fibroblasts (NHLFs) and is activated by polyamines upregulated in IPF. (**A**) Representative image of CaSR expression in NHLF (green), nuclei (blue). Scale bar: 100 µm. (**B**) Summary data of intracellular calcium levels ([Ca^2+^]_i_) in NHLF in response to: (**Ci**) 5 mM Ca^2+^ (divalent cation); (**Di**) 5 mM L-ornithine (basic amino acid); and (**Ei**) 1 mM spermine (polyamine). (**B**,**Cii**–**Eii**) Treatment with a CaSR negative allosteric modulator (CaSR NAM) prevents these increases in [Ca^2+^]_i_. (**C**–**E**) Representative traces of NHLF [Ca^2+^]_i_ response to CaSR activators. Summary data are presented as mean ± SD. Statistical analysis was performed using one-way ANOVA (with Sidak’s post hoc test); *** *p* < 0.001, **** *p* < 0.0001. 5 mM Ca^2+^ (*n* = 3–6; 108 cells), 5 mM L-ornithine (*n* = 5–6; 109 cells), and 1 mM spermine (*n* = 3; 39 cells). *n* = 3–6 independent experiments. CaSR negative allosteric modulator: NAM, NPS2143 (1 μM).

Since fibroblasts are key mediators of the pro-fibrotic response in IPF and intracellular Ca^2+^ signaling underpins several pro-fibrotic processes [56], we explored the ability of polyamines to activate the CaSR in NHLFs. Protein expression of CaSR in untreated NHLFs was confirmed by immunofluorescence (Figure 2A). The functionality of this CaSR expression was then confirmed by observing the response of these fibroblasts to increased [Ca^2+^]_o_ from baseline 1.2 mM to 5 mM (Figure 2B,Ci). Figure 2Ci shows that, as expected, increasing [Ca^2+^]_o_ evoked an increase in [Ca^2+^]_i_, an effect that was abrogated by a CaSR NAM (Figure 2B,Cii).

As seen in Figure 2Di,Ei, both 5 mM ornithine (basic amino acid; Figure 2Di) and 1 mM spermine (polyamine; Figure 2Ei) evoked a significant rise in [Ca^2+^]_i_ from baseline, effects that were again abolished in the presence of NAM (NPS2143, 1 µM; *p* = 0.0001 and *p* = 0.0005, respectively; Figure 2B,Dii,Eii). These results are consistent with the hypotheses that NHLFs express functional CaSR, which can be activated by arginine pathway metabolites, and this effect is abolished by the CaSR NAM, NPS2143.

### 3.3. NHLFs Treated with TGFβ1 Display a Pro-Fibrotic Genetic Profile and Co-Treatment with CaSR NAM Alters TGFβ1-Mediated Changes

TGFβ1 is used in vitro to induce fibrotic phenotypes in fibroblasts of various origins [57]. We used RNA sequencing to assess the suitability of TGFβ1 treatment of NHLFs as an in vitro model of IPF and examined the effects of the CaSR NAM, NPS2143, on relevant biological pathways.

**Figure 3:**
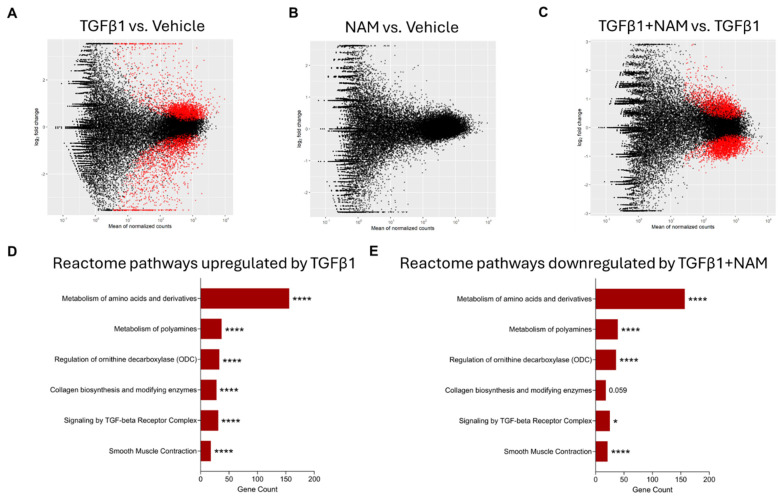
Enrichment analysis of differentially expressed genes (DEGs) from RNA sequencing (RNAseq) in primary human lung fibroblasts treated with vehicle (0.01% DMSO), CaSR NAM (1 μM NPS2143), TGFβ1 (5 ng/mL), and TGFβ1+ CaSR NAM for 72 h. The MA plots show the log_2_-fold change between experimental groups (M) against the mean expression across all the samples (**A**) for each gene. Statistically significant differentially expressed genes (DEGs) for each comparison are shown in red. The x-axis shows the mean of normalized gene counts; the y-axis shows log_2_ fold-changes in expression (positive values represent upregulated genes, while negative values denote downregulation). (**A**) MA plot of RNAseq showing the DEGs in TGFβ1 treated cells relative to unstimulated cells (vehicle control) cells. Red dots represent statistically significant DEGs (Down-DEGs: 1999; Up-DEGs: 2757), while black dots represent non-significantly altered genes. (**B**) No significant difference in gene expression was observed when the CaSR NAM-treated cells were compared with vehicle control cells. (**C**) MA plot showing the DEGs in cells co-treated with TGFβ1 + CaSR NAM relative to TGFβ1-treated cells. Red dots represent statistically significant DEGs (Down-DEG: 2608; Up-DEG: 2150). (**D**,**E**) Annotation of key Reactome pathways based on the enrichment analysis of the statistically significant DEGs. (**D**) Key Reactome pathways upregulated by TGFβ1 relative to vehicle control. (**E**) Key Reactome pathways downregulated by CaSR NAM in the presence of the fibrotic stimulus, TGFβ1, relative to TGFβ1 treatment alone. Benjamini–Hochberg *p*-value adjustment was performed for all statistical tests; level of controlled false positive rate was set to 0.05. * *p* < 0.05, **** *p* < 0.0001. *N* = 3 donors. NAM: Calcium-sensing receptor (CaSR) Negative Allosteric Modulator treatment; TGFβ1: Transforming growth factor β1 (TGFβ1) treatment; TGFβ1 + NAM: TGFβ1 and CaSR Negative Allosteric Modulator co-treatment.

TGFβ1 treatment significantly differentially expressed 4756 genes compared to unstimulated fibroblasts (vehicle control), with 2757 genes upregulated and 1999 genes downregulated (Figure 3A). Treating the fibroblasts with NAM alone did not induce any significant changes in gene expression (Figure 3B). Co-treating NHLFs with TGFβ1 and NAM significantly upregulated 2150 genes whilst downregulating 2608 genes when compared to TGFβ1-treated fibroblasts (Figure 3C).

KEGG enrichment analysis of differentially expressed genes showed that TGFβ1 stimulation upregulated pathways associated with metabolic processes (such as metabolism of amino acids and derivatives, and polyamines), ECM organization, collagen biosynthesis, assembly and degradation, TGFβ signaling, and smooth muscle contraction (Figure S4). Co-treatment of fibroblasts with TGFβ1 and CaSR NAM downregulated pathways associated with amino acid and polyamine metabolism, collagen biosynthesis, TGFβ signaling, and smooth muscle contraction.

### 3.4. CaSR Regulates Polyamine Gene Expression in TGFβ1-Activated NHLF

In the presence of fibrotic stimuli, fibroblasts rely on several metabolic pathways to provide substrates (such as non-essential amino acids and polyamines) required for collagen synthesis [19,58] and growth [59]. We used RNA sequencing to examine the effects of TGFβ1 and the CaSR NAM, NPS2143, on polyamine metabolism gene expression.

**Figure 4:**
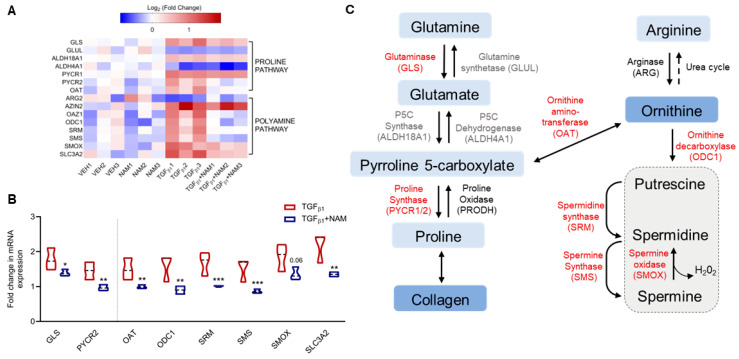
TGFβ1 upregulates genes involved in proline and polyamine metabolism, an effect abrogated by the CaSR NAM, NPS2143, in normal human lung fibroblasts. Fibroblasts were treated with vehicle (0.01% DMSO), CaSR NAM (1 μM NPS2143), TGFβ1 (5 ng/mL), and TGFβ1 + CaSR NAM for 72 h. (**A**) Heatmap generated from RNA sequencing data showing the log_2_ ratio of differentially expressed genes (versus vehicle) implicated in these metabolic pathways. (**B**) RNA sequencing data show exogenous TGFβ1 treatment upregulates genes associated with proline synthesis and polyamine metabolism, while co-treatment with the CaSR NAM, NPS2143 downregulates gene expression, except for *GLUL*, *ALDH4A1*, *ARG2*, and *ALDH18A1*. (**C**) CaSR NAM restores TGFβ1-induced increase in polyamine metabolism to baseline. The genes in red are upregulated by TGFβ1 and downregulated by TGFβ1 and CaSR NAM co-treatment; genes in black were differentially expressed in the presence of TGFβ1, but CaSR NAM co-treatment had no effect on their expression; genes in grey were not affected by either treatment. Genes encoding glutaminase: *GLS*, glutamine synthase: *GLUL,* pyrroline 5-carboxylate synthase: *ALDH18A1*, pyrroline 5-carboxylate dehydrogenase: *ALDH4A1,* pyrroline 5-carboxylate reductase/proline synthase: *PYCR*, proline oxidase: *PRODH*, arginase: *ARG*, ornithine aminotransferase: *OAT*, antizyme inhibitor: *AZIN,* antizyme: *OAZ1*, ornithine decarboxylase: *ODC*, spermidine synthase: *SRM*, spermine synthase: *SMS*, and spermine oxidase: *SMOX,* polyamine transporter-Solute Carrier Family 3 Member 2 (*SLC3A2*) are involved in these pathways. Data in the violin plots represent median fold change, truncated at minimum and maximum values. Benjamini–Hochberg *p*-value adjustment was performed for all statistical tests; level of controlled false positive rate was set to 0.05. * *p* < 0.05, ** *p* < 0.01, *** *p* < 0.001. *N* = 3 donors. CaSR negative allosteric modulator (NAM); NPS2143 (1 μM); Vehicle (VEH).

TGFβ1 increased the expression of several amino acid and polyamine metabolic genes (Figure 4A,B) compared with the vehicle control. A panel of the full gene set is presented in Figure S5. Exposure of NHLF to TGFβ1 resulted in a significant increase in the expression of key genes involved in the proline–polyamine pathway, namely, glutaminase (*GLS)*, pyrroline 5-carboxylate synthase (*ALDH18A1*), and ornithine aminotransferase (*OAT*), with concomitant downregulation of glutamine synthase (*GLUL*) and pyrroline 5-carboxylate dehydrogenase (*ALDH4A1*), which favor the conversion of pyrroline 5-carboxylate back into glutamine (Figure 4). In the presence of TGFβ1, CaSR NAM decreased *GLS* expression (*p_adj_* value *=* 0.02) and restored baseline expression of pyrroline 5-carboxylate reductase (*PYCR2; p_adj_* value *=* 0.003) and *OAT* (*p_adj_* value *=* 0.003) (Figure 4B).

Since ornithine is a precursor for polyamine synthesis, we explored this pathway in more detail. In the presence of TGFβ1, the NHLF exhibited a minimum 1.5-fold increase in expression of genes encoding the enzymes in the polyamine pathway, except for arginase (*ARG2*), which was not significantly altered (fold change = −0.5; *p_adj_* value > 0.05). Baseline expression of these genes was restored in the presence of the CaSR NAM and TGFβ1: ornithine decarboxylase 1 (*ODC1*; *p_adj_* value *=* 0.002), ornithine decarboxylase antizyme 1 (*OAZ1; p_adj_* value *=* 0.0002), spermidine synthase (*SRM; p_adj_* value *=* 0.0003), and spermine synthase (*SMS; p_adj_* value *=* 0.0001) (Figure 4A,B).

The expression of mRNA encoding spermine oxidase (*SMOX*), which converts spermine back to spermidine, was also significantly elevated in the presence of TGFβ1 (fold change = 1.9; *p_adj_* value = 0.0002), as was that of mRNA encoding antizyme inhibitor (*AZIN2*) (fold change = 1.4; *p_adj_* value < 0.0001; Figure 4A,B), which positively regulates ornithine decarboxylase activity. Co-treatment with the CaSR NAM restored the baseline expression of SMOX (*p_adj_* value = 0.06); however, AZIN2 expression was unchanged. Expression of the polyamine transporter (*SLC3A2*) increased with TGFβ1 treatment (*p_adj_* value < 0.0001; Figure 4A,B), while co-treatment with CaSR NAM reduced SLC3A2 gene expression (*p_adj_* value = 0.002; Figure 4B). Treatment with CaSR NAM alone had no significant effect on the expression of the aforementioned genes (Figure S5).

### 3.5. CaSR Regulates Pro-Fibrotic Gene Expression in TGFβ1-Activated NHLF

**Figure 5:**
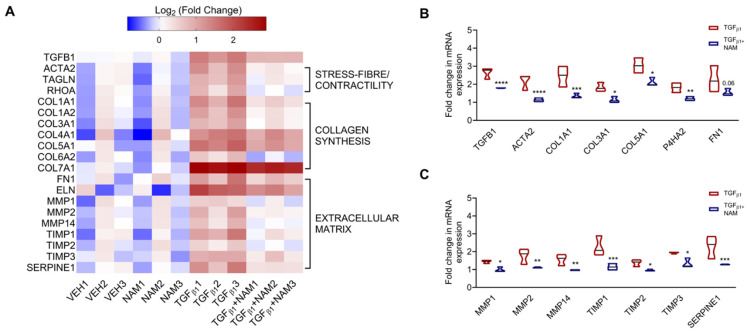
CaSR negative allosteric modulator, NPS2143, attenuates TGFβ1-induced upregulation of pro-fibrotic genes in normal human lung fibroblasts. NHLFs were treated with vehicle (0.01% DMSO), CaSR NAM (1 μM NPS2143), TGFβ1 (5 ng/mL), and TGFβ1 + CaSR NAM for 72 h. (**A**) Heatmap generated from RNA sequencing data showing the log_2_ ratio of differentially expressed genes (versus vehicle). Exogenous TGFβ1 application upregulates genes related to fibroblast contractility, collagen synthesis, and extracellular matrix (ECM) remodeling. (**B**) CaSR NAM reduces the expression of genes associated with fibroblast contractility, collagen synthesis and assembly; and (**C**) ECM remodeling and maintenance. Genes encoding Transforming Growth Factor-β1: *TGFB1*, α-smooth muscle actin: *ACTA2,* smooth muscle-22α: *TAGLN*, ras homolog family member A: *RHOA,* collagen: *COL*, prolyl 4-hydroxylase, alpha polypeptide II: *P4HA2,* fibronectin: *FN1*, elastin: *ELN*, matrix-metalloproteinases: *MMP*, tissue inhibitors of metalloproteinases: *TIMP*, and serine protease inhibitor E1: *SERPINE1* are involved in these pathways. Data in the violin plots represent median fold change, truncated at minimum and maximum values. Benjamini–Hochberg *p*-value adjustment was performed in all statistical tests; level of controlled false positive rate was set to 0.05. * *p_adj_* <0.05, ** *p_adj_* <0.01, *** *p_adj_* <0.001, **** *p_adj_* <0.0001. *N* = 3 donors. CaSR negative allosteric modulator: NAM, NPS2143 (1 μM).

Cytoskeletal changes and excessive collagen and ECM deposition are central to IPF pathophysiology [60]. Our RNAseq data demonstrated that genes implicated in these pathways are enriched by TGFβ1 treatment in vitro (Figure 5A). Exogenous TGFβ1 treatment upregulated TGFB1 gene expression (fold change = 1.4; *p_adj_* value *<* 0.0001), while co-treatment with CaSR NAM reduced its expression (fold change = −0.6; *p_adj_* value *<* 0.0001; Figure 5A,B). Expression of the cytoskeletal gene encoding α-smooth muscle actin (*ACTA2*) was doubled in the presence of TGFβ1 (*p_adj_* value < 0.0001; Figure 5B). Co-treatment with CaSR NAM completely abolished this pro-fibrotic effect (*p_adj_* < 0.0001; Figure 5B). Several collagen subtypes and genes that maintain collagen fiber integrity and stability were upregulated by TGFβ1 application, e.g., collagens *COL1A1, COL3A1, COL5A1,* and prolyl 4-hydroxylase, alpha polypeptide II *(P4HA2)* (*p_adj_* value < 0.05; Figure 5A,B). *COL4A1* and *COL7A1*, which are involved in basement membrane integrity, were also upregulated by TGFβ1. Lastly, key ECM-related genes were upregulated by pro-fibrotic stimuli, including *COL6A2,* fibronectin (*FN1*), elastin (*ELN*), matrix-metalloproteinases (MMP1, MMP2, MMP14), tissue inhibitors of metalloproteinases (TIMP1, TIMP2, TIMP3), and serine protease inhibitor E1 (*SERPINE1*) (*p_adj_* value < 0.05; Figure 5A and Figure S6). Co-treatment with CaSR NAM reduced the expression of these genes except COL4A1 (*p_adj_* value < 0.05; Figure 5A,B).

Importantly, Figure 4 and Figure 5 show that the gene expression profile of NHLFs treated with CaSR NAM alone was not statistically different from unstimulated cells, i.e., vehicle control, indicating that the CaSR NAM does not have an effect without TGFβ1 stimulation in our model.

### 3.6. Effects of TGFβ1 on CaSR Expression in Normal and IPF Primary Lung Fibroblasts

**Figure 6:**
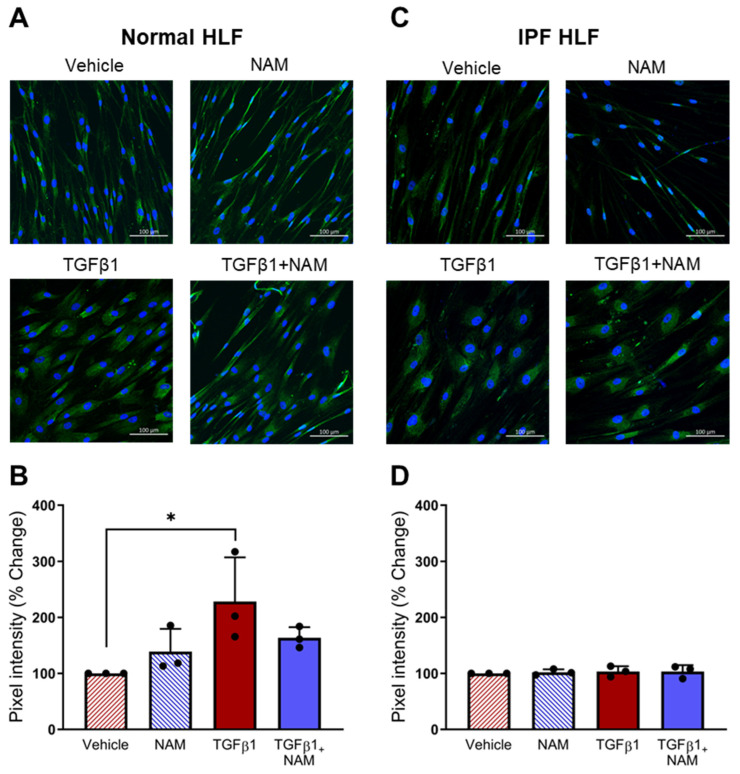
CaSR is expressed in normal human lung fibroblasts (NHLFs) and IPF human lung fibroblasts (IPF HLFs). (**A**) Representative images showing CaSR expression when NHLFs were treated with vehicle (0.01% DMSO), CaSR NAM (1 μM NPS2143), TGFβ1 (5 ng/mL), and TGFβ1 + CaSR NAM for 72 h. (**B**) Image quantification analysis with bespoke software, StrataQuest, shows that TGFβ1 treatment increases CaSR expression in NHLFs. (**C**) Representative images showing CaSR expression in IPF HLFs in the same treatment conditions. (**D**) Image quantification with StrataQuest shows CaSR expression is maintained in IPF HLFs in all treatment conditions. Data are presented as mean ± SD. Statistical analysis was performed using one-way ANOVA (with Sidak’s post hoc test); * *p* < 0.05. *N* = 3 donors; *n* = 3 independent experiments. Scale bar: 100 µm. CaSR negative allosteric modulator: NAM.

Next, we investigated the effects of TGFβ1 and NAM on CaSR protein expression in normal and IPF HLFs (Figure 6). In the fibroblasts from normal control subjects, CaSR expression doubled in the presence of TGFβ1 (*p* = 0.03), while receptor expression was reduced when co-treated with CaSR NAM. In IPF HLFs, CaSR expression was maintained at a comparable level across all treatment conditions. These results suggest that CaSR expression is upregulated in response to pro-fibrotic stimulus in healthy HLF, an effect prevented by a CaSR NAM. Baseline CaSR expression was unaffected by NAM treatment in the absence of TGFβ1 co-stimulation.

### 3.7. TGFβ1 Upregulates Polyamine Production in Normal and IPF HLF, and CaSR Antagonism Abrogates These Responses

Given that polyamine metabolites are enriched in IPF saliva samples and their gene transcripts are increased in the presence of pro-fibrotic stimuli, we investigated whether exposure of normal and IPF fibroblasts to TGFβ1 could directly increase the expression of polyamines.

**Figure 7:**
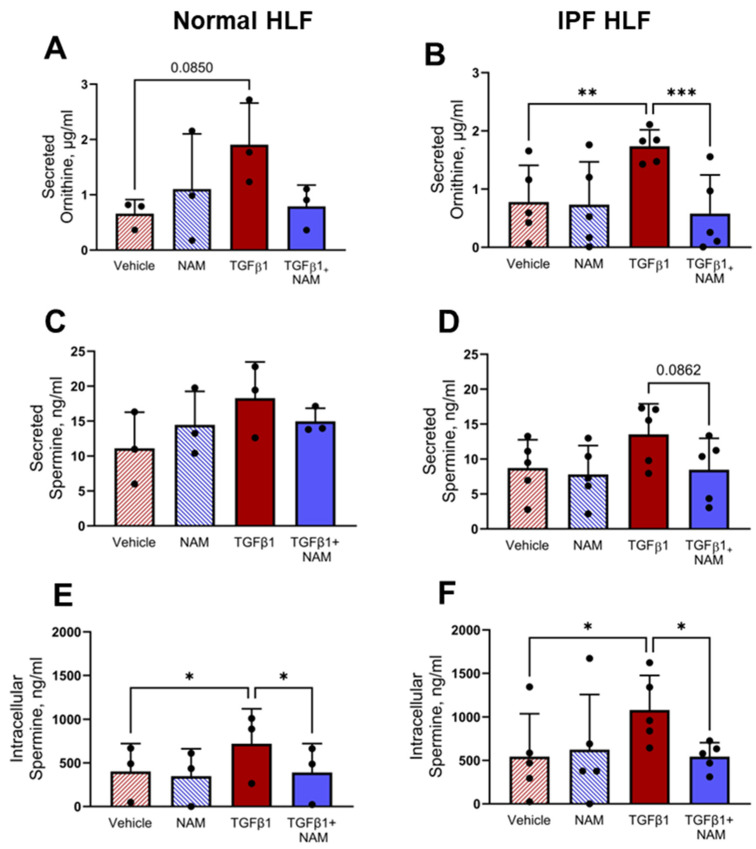
TGFβ1-induced polyamine expression is abrogated by CaSR NAM in normal and IPF human lung fibroblasts (HLFs). (**A**,**B**) Ornithine secretion by normal HLFs and IPF HLFs is increased by TGFβ1 treatment, and the response is attenuated in the presence of CaSR NAM. (**C**,**D**) Low concentration of spermine is secreted by normal HLFs and IPF HLFs, with levels remaining relatively unchanged across the treatment conditions. (**E**,**F**) Intracellular spermine concentration is increased by exogenous TGFβ1 treatment in normal HLFs and IPF HLFs; co-treatment with CaSR NAM restores vehicle control levels of the polyamine. Data are presented as mean ± SD. Statistical analysis was performed using one-way ANOVA (with Sidak’s post hoc test); * *p* < 0.05, ** *p* < 0.01, *** *p* < 0.001. *N* = 3 donors; *n* = 3–6 independent experiments. CaSR negative allosteric modulator: NAM, NPS2143 (1 μM).

Exogenous treatment of NHLFs and IPF HLFs with TGFβ1 induced ornithine secretion; CaSR NAM co-treatment reduced ornithine secretion in IPF fibroblasts by 65% (*p* = 0.0003) (Figure 7A,B). Due to the low concentration of spermine secreted in all treatment conditions (Figure 7C,D), we investigated the intracellular concentration of spermine in normal and IPF HLFs. TGFβ1 increased spermine concentration in both normal and diseased fibroblasts (*p* < 0.05). In both circumstances, co-treatment with CaSR NAM restored baseline levels of spermine (*p* < 0.05; Figure 7E,F).

### 3.8. CaSR Antagonism Reduces TGFβ1 Pro-Fibrotic Responses in IPF Lung Fibroblasts

**Figure 8:**
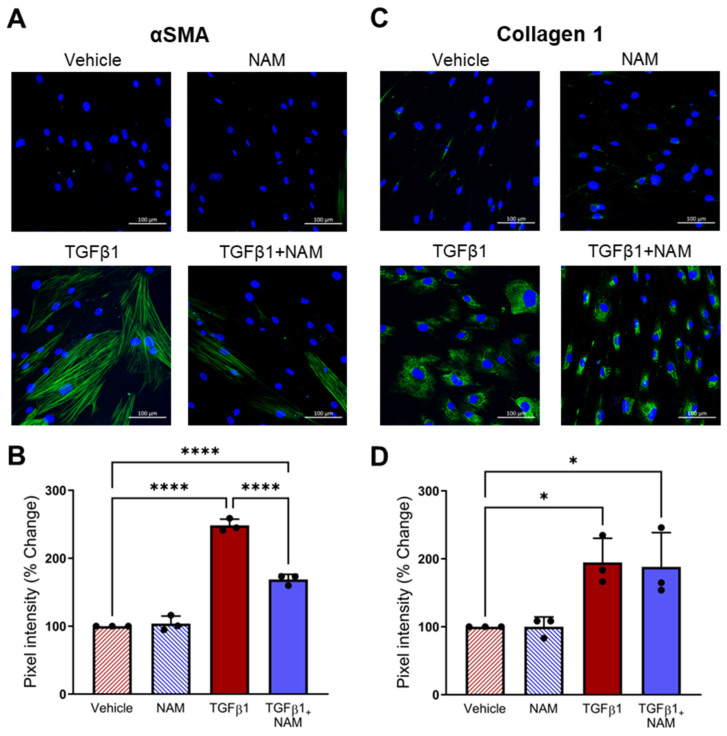
CaSR NAM reduces TGFβ1-induced pro-fibrotic changes in IPF human lung fibroblasts (HLFs). IPF HLFs were treated with vehicle (0.01% DMSO), CaSR NAM (1 μM NPS2143), TGFβ1 (5 ng/mL), and TGFβ1 + CaSR NAM for 72 h. (**A**) Representative images showing that TGFβ1 treatment increases alpha smooth muscle actin (αSMA) expression and stress-fibre formation (green), a response abrogated by the CaSR NAM. (**B**) Image quantification analysis shows that TGFβ1-induced increase in αSMA expression is halved in the presence of CaSR NAM. (**C**) Representative images showing diffuse Collagen 1 expression in IPF HLFs treated with TGFβ1. Co-treatment with CaSR NAM appears to reduce cell area (see Figure S7). (**D**) Image quantification confirms increased collagen 1 expression in IPF HLFs treated with TGFβ1. Data are presented as mean ± SD. Statistical analysis was performed using one-way ANOVA (with Sidak’s post hoc test); * *p* < 0.05, **** *p* < 0.0001. *N* = 3 donors; *n* = 3 independent experiments. Scale bar: 100 µm. CaSR negative allosteric modulator: NAM.

We also assessed the expression of pro-fibrotic markers implicated in IPF. TGFβ1 treatment increased αSMA expression (*p* < 0.0001) and collagen expression (*p* = 0.03), compared with vehicle control (Figure 8). CaSR NAM co-treatment reduced αSMA protein expression (*p* < 0.0001; Figure 8A,B). While CaSR NAM treatment did not reduce total collagen expression, it altered its expression pattern from diffuse to concentrated (Figure 8C,D) and modified the morphology of the cells from large and extended (associated with more secretory and contractile functions) to smaller, spindle-shaped morphology, typical of inactive fibroblasts [61] (Figure S7).

## 4. Discussion

In recent years, arginine pathway metabolites have emerged as important biomarkers in the pathogenesis of lung disease [62,63,64,65,66], but whether polyamines actively participate in IPF pathogenesis or are mainly bystanders remains unclear. Our studies show that: (1) polyamine levels are increased in the saliva of IPF patients; (2) polyamines directly act at the CaSR, a receptor expressed in normal and IPF human lung fibroblasts, leading to a rise in intracellular calcium concentration; (3) TGFβ1 upregulates the expression of both polyamines and CaSR in NHLFs, and polyamines only in IPF fibroblasts; and (4) CaSR inhibition with NAM abrogates the key effects of pro-fibrotic stimuli in both normal and IPF HLFs, suggesting that the CaSR and NAM might hold therapeutic potential for the treatment of IPF. Furthermore, our studies indicate that the levels of expression of arginine pathway-derived polyamines could provide valuable non-invasive tools in the diagnosis and management of IPF.

Our findings show that the saliva of IPF patients is enriched in arginine pathway metabolites compared to healthy controls. This aligns with studies from other groups that report increased levels of circulating ornithine and ornithine-derived metabolites, putrescine and spermidine in IPF lung tissue [18,67]. These natural polyamines are established activators of the CaSR [28], which, in many cell types, elicit an increase in intracellular calcium [Ca^2+^]_i_ via the activation of G protein signaling pathways [24]. Here, our results demonstrate that CaSR is functionally expressed by normal human lung fibroblasts, as confirmed by the use of the classical ligand for the receptor, calcium ions, which induced robust Ca^2+^ mobilization in HLFs. This response was abolished by co-treatment with NAM. Pertinently, this study shows that ornithine and spermine also activate the CaSR in fibroblasts, evoking similar increases in [Ca^2+^]_i_. Since increases in [Ca^2+^]_i_ underpin key fibrotic processes, such as activating genes responsible for the production of TGFβ, collagen, and α-smooth muscle actin (αSMA) in fibroblasts [56], our findings suggest a potential pathophysiological interplay between polyamines, the CaSR and Ca^2+^_i_, which may contribute to IPF development.

Using TGFβ1-treated human lung fibroblasts as an in vitro model of IPF, we have shown that pro-fibrotic stimuli upregulate the expression of gene pathways associated with increases of cytosolic calcium concentrations and increased metabolism of amino acids. Polyamine expression and secretion are also increased in the presence of pro-fibrotic stimuli, which is consistent with our metabolomic data from patient saliva. Although ARG2 (encoding arginase) expression remained unchanged in our model, we observed significant alterations in the key genes involved in metabolizing all the polyamines in this pathway, i.e., ornithine, putrescine, spermidine, and spermine, which are increased in IPF patients [18]. These findings suggest that arginase activity may still be relevant, and future studies could investigate this at the protein level to clarify its potential role in this context. An alternative source of polyamines precursor, ornithine, can be synthetized from glutamate, and upregulated OAT (encoding ornithine aminotransferase) is likely to fulfill this role. Notably, in the pro-fibrotic context, CaSR negative allosteric modulation abrogates this increase in polyamines and the polyamine exporter, SLC3A2, at the transcriptomic level in lung fibroblasts from healthy subjects, and at the molecular level in lung fibroblasts from patients with IPF. Furthermore, treating fibroblasts with NAM on its own did not result in differential gene expression, which is consistent with NAMs acting as allosteric modulators and being inactive in the absence of ligands [68]. Since polyamines play an essential role in cellular growth, survival, and proliferation, they are attractive therapeutic targets not only in such highly proliferative diseases as cancer [69], but also in several unrelated lung diseases, such as asthma [24], pulmonary hypertension [70], and respiratory viral infections [66].

In asthma and pulmonary hypertension, direct activation of CaSR with the polyamines and/or polycationic proteins increases cytosolic calcium signaling, smooth muscle cell proliferation, and hyperresponsiveness, all of which are abolished by genetic or pharmacological inhibition of CaSR [24,35,70]. Notably, increased levels of polyamines are essential for virus–host interaction, viral invasion, and replication [66,71]. In addition, several pathogenic bacteria use polyamines to avoid host immune defenses and improve survival [72]. Several studies have also shown that cells infected by viruses associated with IPF exhibit increased glutamine utilization [73,74,75,76]. Upregulation of the glutamine metabolic pathway is crucial for collagen synthesis in IPF [17], with lower circulating levels of glutamine being associated with an increased risk of IPF [77]. Arguably, this decrease in circulating glutamine levels could be a result of the upregulation of downstream metabolic pathways such as proline and polyamine metabolism. Our results demonstrate that treatment with TGFβ1 upregulates genes associated with the glutamine degradation pathway, such as glutaminase, pyrroline 5-carboxylate synthase, and ornithine aminotransferase, while downregulating genes associated with glutamine synthesis, such as glutamine synthase and pyrroline 5-carboxylate dehydrogenase, thus favoring proline and ornithine biosynthesis instead of glutamine. Importantly, treatment with CaSR NAM restored the baseline expression of these genes in our in vitro IPF model, suggesting a potential role for the abovementioned metabolic dysregulation in IPF through the activation of CaSR. Furthermore, the polyamine-CaSR pathway might augment pro-fibrotic processes and facilitate infections, leading to a vicious cycle.

Fibrotic remodeling involves the perturbation of the balance between ECM deposition and degradation, with the scales tipped in favor of deposition [78]. Furthermore, TGFβ1 acts as a ‘master switch’ for the secretion of collagen and other ECM proteins from αSMA-expressing myofibroblasts [79]. Our RNAseq data demonstrates that exogenous TGFβ1 autoregulates its expression at the transcriptomic level, which can lead to an amplification of the pro-fibrotic signal. Several TGFβ1-mediated pathways are implicated in αSMA stress fiber formation, including ERK, Akt, and Rho-kinase activation [80], which are all signaling pathways downstream of the CaSR. The RNAseq data from our in vitro model of IPF demonstrate upregulation of TGFβ1-signaling pathways, collagen biosynthetic pathways, and genes implicated in smooth muscle contractility. Notably, the negative allosteric modulator of the CaSR, NPS2143, attenuated the upregulation of all the above pathways, targeting the key pathological hallmarks of IPF: transition to myofibroblast and contractility, collagen synthesis, ECM regulation, and altered cell morphology, suggesting the receptor plays a role in IPF pathogenesis.

Maintaining the structural integrity of the ECM also involves matrix proteinases, MMPs, and their inhibitor proteins, TIMPs, both of which are known to be increased in IPF [81]. Our results highlight enhanced expression of MMP1, responsible for fibrillar collagen, fibronectin, and elastin degradation, and MMP2, which participates in the degradation of the basement membrane [82]. The upregulation of MMPs (especially MMP1) may contribute to cleavage and activation of a plethora of cytokines, including pro-TNFα, pro-1L-1β, and IGFBP3 [83,84], which play a key role in IPF. Interestingly, extracellular MMP1 and 2 also activate their pro-peptides, creating a positive feedback loop, whilst intracellular MMP1 accumulates during mitosis, and inhibiting this enzyme resulted in a faster induction of apoptosis compared to untreated cells, suggesting that it might be important in maintaining the balance between cell division and cell death [85]. Lastly, the concomitant increase in the gene transcripts of protease inhibitors, TIMP1, TIMP3, and SERPINE1, suggest that CaSR may facilitate a profibrotic environment promoting a non-degrading collagen-rich ECM, which is a defining feature of human and animal models of lung fibrosis [86,87].

## 5. Conclusions

Our findings demonstrate that CaSR contributes to the pro-fibrotic responses in human lung fibroblasts, suggesting novel therapeutic tools for IPF management. Given that polyamines, elevated in the saliva of IPF patients and in the in vitro model of IPF, are the physiological CaSR ligands, this study also provides a potential mechanistic link between the receptor and the TGFβ1-induced upregulation of polyamine metabolism. Notably, as early diagnosis remains critical, our findings, together with patient preference for non-invasive disease monitoring methods, provide a rationale to explore whether polyamines in patient saliva could be used as a viable biomarker for disease detection, patient stratification, and prediction of prophylactic and therapeutic responses to CaSR NAM treatment for IPF.

## Data Availability

The original contributions presented in this study are included in the article/Supplementary Material. Further inquiries can be directed to the corresponding author(s). Bioinformatics data presented in this study are openly available in NCBI Sequence Read Archive (SRA) at https://www.ncbi.nlm.nih.gov/sra/ (accessed on 18 March 2025) or accession number: PRJNA1213828.

## Supplementary Materials

The following supporting information can be downloaded at: https://www.mdpi.com/article/10.3390/biom15040509/s1, Table S1. Clinical and histopathological characteristics of metabolomic samples. Figure S1. Representative images showing immunofluorescence negative control staining; Figure S2. Representative images of in vitro immunofluorescent mask labelling protocol using StrataQuest. Figure S3. Metabolomic fingerprint of idiopathic pulmonary fibrosis (IPF) patient saliva samples. Figure S4. Annotation of key Reactome pathways upregulated by TGFβ1 in human primary lung fibroblasts based on the enrichment analysis of the obtained DEGs. Figure S5. CaSR negative allosteric modulator, NPS2143 attenuates TGFβ1-induced up-regulation of genes associated with amino acid metabolism and polyamines in normal human lung fibroblasts. Figure S6. CaSR negative allosteric modulator, NPS2143 attenuates TGFβ1-induced up-regulation of pro-fibrotic genes in normal human lung fibroblasts. Figure S7. CaSR NAM reduces TGFβ1-induced increase in cell area in IPF lung fibroblasts.
