# Supplementary material for: Calcium-Sensing Receptor as a Novel Target for the Treatment of Idiopathic Pulmonary Fibrosis
Source: Biomolecules. 2025 Apr 1;15(4):509. doi: 10.3390/biom15040509 (PMC12025166; doi:10.3390/biom15040509)
Supplement: Supplementary file 1 [file biomolecules-15-00509-s001.zip › biomolecules-3384382-supplementary.pdf]

biomolecules

MDPI

Supplementary Materials

Table S1. Clinical and histopathological characteristics of metabolomic samples.

Variables	Healthy Control	Idiopathic Pulmonary Fibrosis
**Age**		
Median	70	82
Range	48–85	73–87
**Sex at birth**		
Male	2	4
Female	4	2
**Smoking status**		
Never smoked	2	2
Ex-smoker	4	4

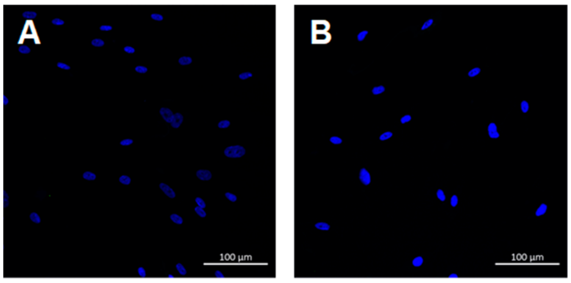

Figure S1. Representative images showing immunofluorescence negative control staining. The methodology involved (**A**) replacing the CaSR antibody with an IgG2a isotype control or (**B**) omitting primary antibodies. Images obtained from primary normal human lung fibroblasts; scale bar: 100 µm.

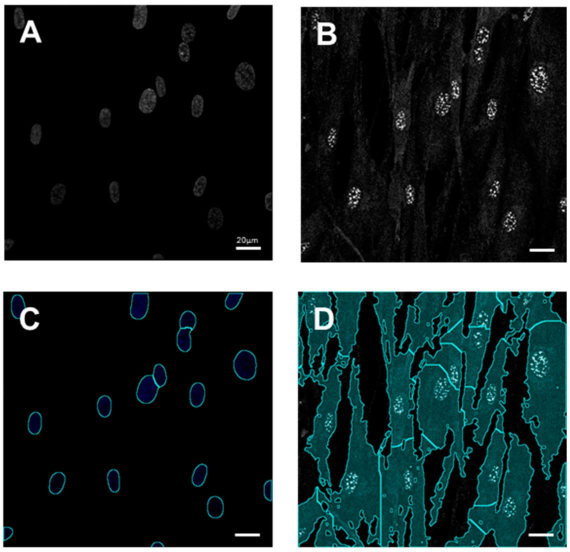

Figure S2. Representative images of *in vitro* immunofluorescent mask labelling protocol using StrataQuest. The figure shows representative images from two wavelengths, (**A**) DAPI and (**B**) 488 in greyscale. High marker presence is indicated by increased "white" pixel intensity while low marker presence appears darker. (**C**) Representative nuclei mask image and (**D**) Representative cell measurement mask image used to determine mean immunoreactivity intensity as a measurement of protein marker expression. Images obtained from primary normal human lung fibroblasts treated with 5 ng/ml TGF-β1; scale bar: 20 µm.

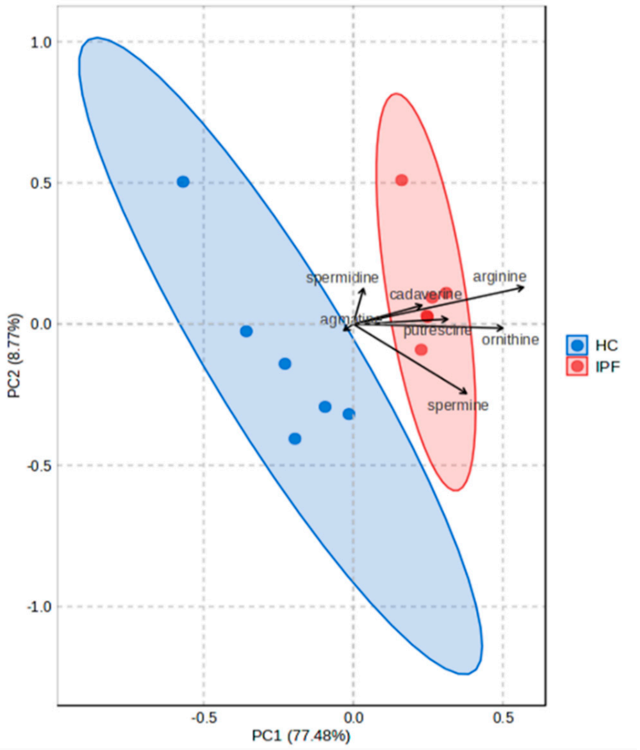

Figure S3. Metabolomic fingerprint of idiopathic pulmonary fibrosis (IPF) patient saliva samples. Principal component analysis (PCA) score plot between the main principal components (PC1 and PC2) shows a tight cluster of the IPF samples based on their metabolic profile compared to controls with a 95% confidence ellipse drawn for each group (shown by the different colors: red control—(HC); blue—IPF). Assessment of PC1 describes 77.48% of the total variation and separates between the control (HC) and IPF cluster. The loading vectors show the importance of charged amino acids (arginine and ornithine) but also the contribution of polyamines in driving the discrimination between HC and IPF clusters. N = 6 controls; 6 IPF patients.

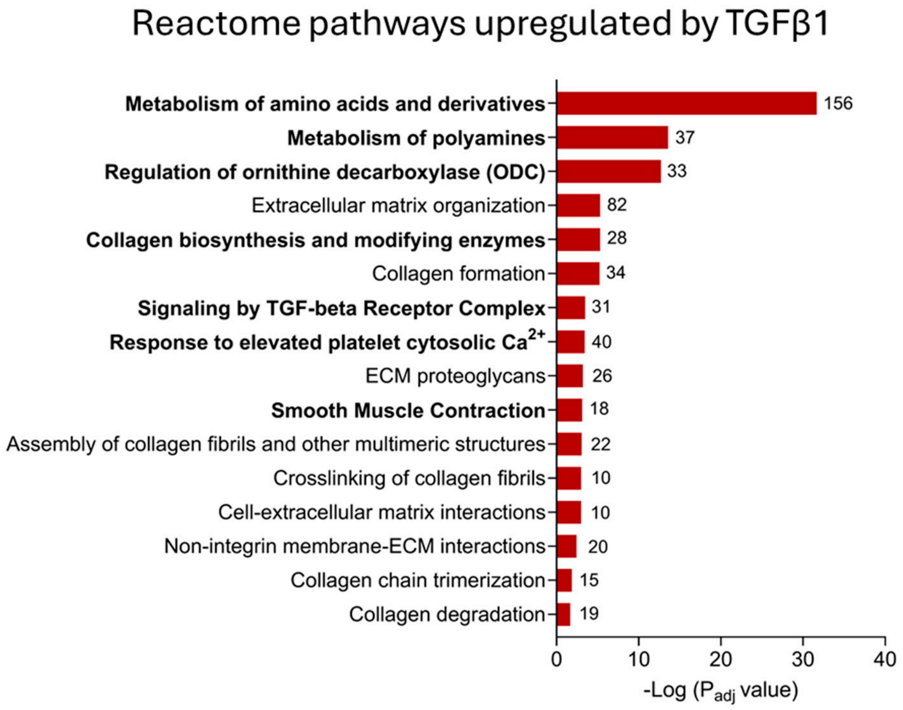

Figure S4. Annotation of key Reactome pathways upregulated by TGFβ1 in human primary lung fibroblasts based on the enrichment analysis of the obtained DEGs. Kyoto Encyclopedia of Genes and Genomes (KEGG) enrichment analysis of the top 5% of differentially expressed genes (DEGs) of fibroblasts treated with TGFβ1 compared with unstimulated cells (vehicle control). The pathways that are also downregulated by TGFβ1 and CaSR NAM co-treatment are highlighted in bold. The enriched pathways are plotted against their –log(adjusted p value), with the most significant p values indicated by the longest bars. Gene counts for each pathway is shown for each bar. p-value adjustment was performed for all statistical tests; level of controlled false positive rate was set to 0.05. N = 3 donors. NAM: CaSR negative allosteric modulator, NPS2143 (1 μM).

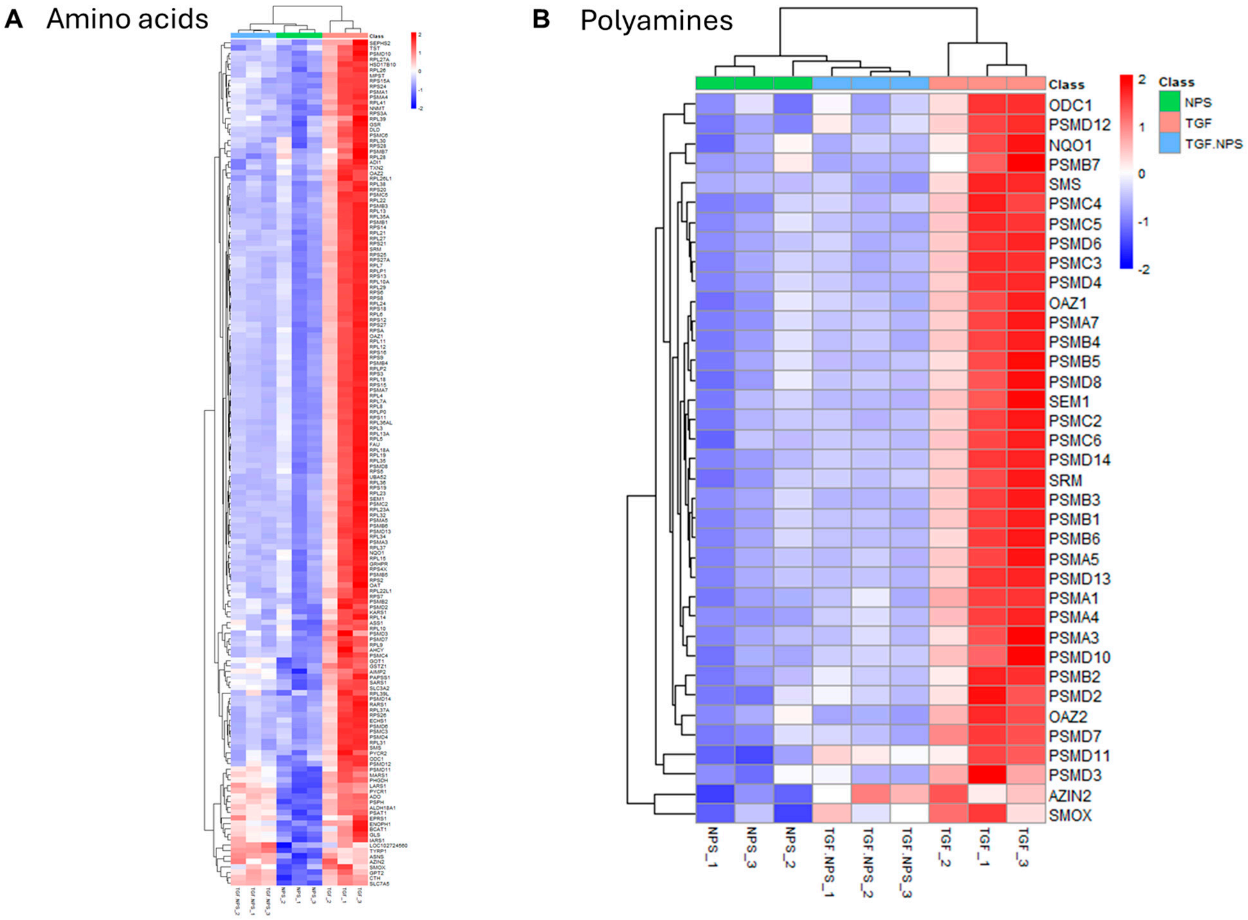

Figure S5. CaSR negative allosteric modulator, NPS2143 attenuates TGFβ1-induced upregulation of genes associated with amino acid metabolism and polyamines in normal human lung fibroblasts. Fibroblasts were treated with vehicle (0.01% DMSO), CaSR NAM (1 μM NPS2143), TGFβ1 (5 ng/mL) and TGFβ1+CaSR NAM for 72 hours. (**A**,**B**) Heatmaps generated from RNA sequencing data showing the $\log_2$ ratio of differentially expressed genes (versus vehicle). Exogenous TGFβ1 application upregulates genes related to metabolism of amino acids and derivatives, metabolism of polyamines and regulation of ornithine decarboxylase. CaSR NAM reduces the expression of key genes associated with these pathways. Benjamini-Hochberg p-value adjustment was performed in all statistical tests; level of controlled false positive rate was set to 0.05. N = 3 donors. CaSR negative allosteric modulator: NAM, NPS2143 (1 μM).

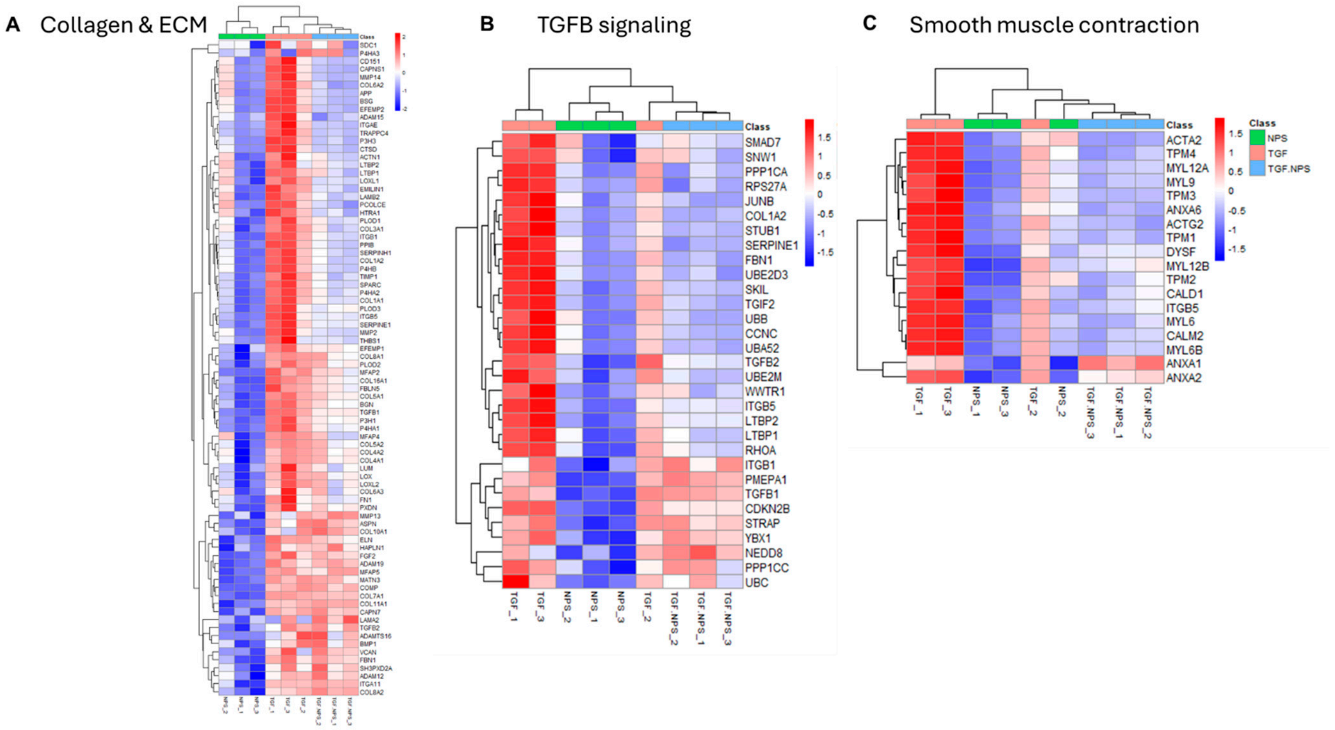

Figure S6. CaSR negative allosteric modulator, NPS2143 attenuates TGFβ1-induced upregulation of pro-fibrotic genes in normal human lung fibroblasts. NHLFs were treated with vehicle (0.01% DMSO), CaSR NAM (1 μM NPS2143), TGFβ1 (5 ng/mL) and TGFβ1+CaSR NAM for 72 hours. (**A–C**) Heatmaps generated from RNA sequencing data showing the $\log_2$ ratio of differentially expressed genes (versus vehicle). Exogenous TGFβ1 application upregulates genes related to collagen synthesis, assembly and degradation, extracellular matrix (ECM) remodeling, TGFβ signaling and contractility. CaSR NAM reduces the expression of key genes associated with these pathways. Benjamini-Hochberg p-value adjustment was performed in all statistical tests; level of controlled false positive rate was set to 0.05. N = 3 donors. CaSR negative allosteric modulator: NAM, NPS2143 (1 μM).

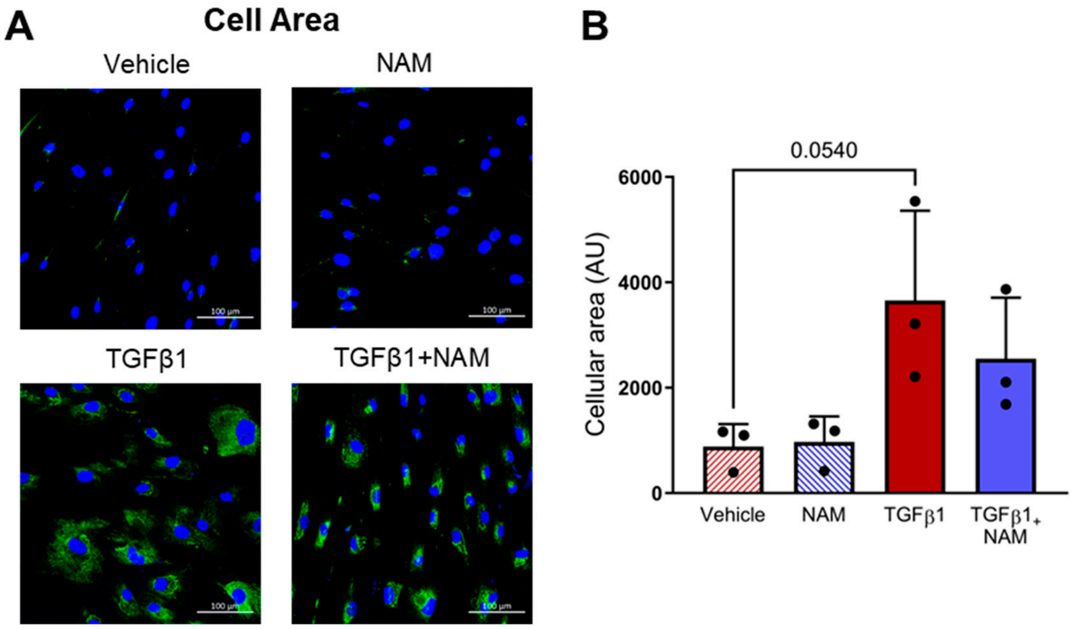

Figure S7. CaSR NAM reduces TGFβ1-induced increase in cell area in IPF lung fibroblasts. IPF HLFs were treated with vehicle (0.01% DMSO), CaSR NAM (1 μM NPS2143), TGFβ1 (5 ng/mL) and TGFβ1+CaSR NAM for 72 hours. (**A**,**B**) Representative images showing Collagen 1 expression in IPF HLFs. TGFβ1 treatment increases cell area while co-treatment with CaSR NAM reduces this response. Data are presented as mean ± SD. Statistical analysis was performed using one-way ANOVA (with Sidak's post hoc test). N = 3 donors; *n* = 3 independent experiments. Scale bar: 100 μm. CaSR negative allosteric modulator: NAM.
